# Intervention of artemisinin in macular edema associated with retinal vein occlusion: A protocol for a systematic review and meta-analysis

**DOI:** 10.1097/MD.0000000000016044

**Published:** 2019-06-21

**Authors:** Jing Xu, Xiaofeng Hao, Bingwen Lu, Jing Ming, Xiaoyu Li, Yixin Qi, Like Xie

**Affiliations:** Department of Ophthalmology, Eye Hospital, China Academy of Chinese Medical Sciences, Beijing, China.

**Keywords:** artemisinin, central retinal thickness, macular edema, optical coherence tomography angiography, retinal vein occlusion

## Abstract

**Background:**

Artemisinin was discovered to be highly effective antimalarial drugs shortly after the isolation of the parent artemisinin in 1971 in China. It is derived from extracts of sweet wormwood (*Artemisia annua*) and are well established for the treatment of malaria. Recently, artemisinin has been shown that it might have therapeutic value for several other diseases. The purpose of this review is to assess the efficacy of artemisinin as a treatment for macular edema associated with retinal vein occlusion.

**Methods and analysis:**

A systematic literature search will be performed in all available databases to quantitatively review eligible studies and identify all relevant data. We will include randomized controlled trials assessing efficacy of artemisinin as a treatment for macular edema associated with retinal vein occlusion. The methodological qualities, including the risk of bias, will be evaluated using the Cochrane risk of bias assessment tool, while confidence in the cumulative evidence will be evaluated using the Grading of Recommendations Assessment, Development and Evaluation (GRADE) approach.

**Ethics and dissemination:**

Ethical approval is not required, as this study is based on the review of published research. This review will be published in a peer-reviewed journal and disseminated both electronically and in print.

**PROSPERO registration number:**

The protocol for this systematic review has been registered on PROSPERO under the number CRD42019131408.

## 1 Introduction

Retinal vein occlusion (RVO) is a prevalent vision-threatening disease,^[1,2]^ which is the second largest retinal vascular disease leading to blindness safter diabetic retinopathy.^[3]^ RVOs are classified based on the site of the occlusion as branch retinal vein occlusion (BRVO), central retinal vein occlusion (CRVO), and hemiretinal vein occlusion. And also it can be further divided into ischemic and non-ischemic types. Macular edema (ME) is a common complication and a primary cause of vision loss in all forms of RVO.^[4,5]^ Early treatment of ME associated with RVO is with better long-term visual outcomes.^[6,7]^

The formation of ME secondary to RVO is mainly due to increased secretion of vascular endothelial growth factor (VEGF). VEGF is an important factor that stimulates the formation of new blood vessels in the choroid and retina.^[8]^ It leads to increased vascular permeability and angiogenesis, aggravating retinal ischemia, and hypoxia.^[9]^ This further aggravate the ME. Anti-VEGF treatment has become the main treatment method for ME secondary to RVO.

At present, anti-VEGF drugs have been widely used with definite clinical efficacy, such as.^[10]^ But there exist many problems. The effects are not long-lasting, requiring repeated injections. And the drugs are expensive and may cause a variety of complications. It is of great significance to find a safer and more effective treatment for ME associated with RVO.

Artemisinin is a sesquiterpenoid lactolide compound containing peroxide group extracted from artemisia annua by Chinese scientists.^[11]^ It not only can resist malaria, but also parasite and tumor, and affect the immune system function and inhibit vascular hyperplasia. Therefore, it is necessary to evaluate the efficacy of artemisinin in the treatment of ME associated with RVO.

## 2 Outcomes

The primary outcomes of this review are visual acuity (VA) and central retinal thickness (CRT). The second outcomes are parameters on optical coherence tomography angiography (OCTA) including the following items: superficial capillary plexus (SCP), deep capillary plexus (DCP), vessel density (VD), foveal avascular zone (FAZ).

## 3 Materials and methods

### 3.1 Study registration

**Figure 1:**
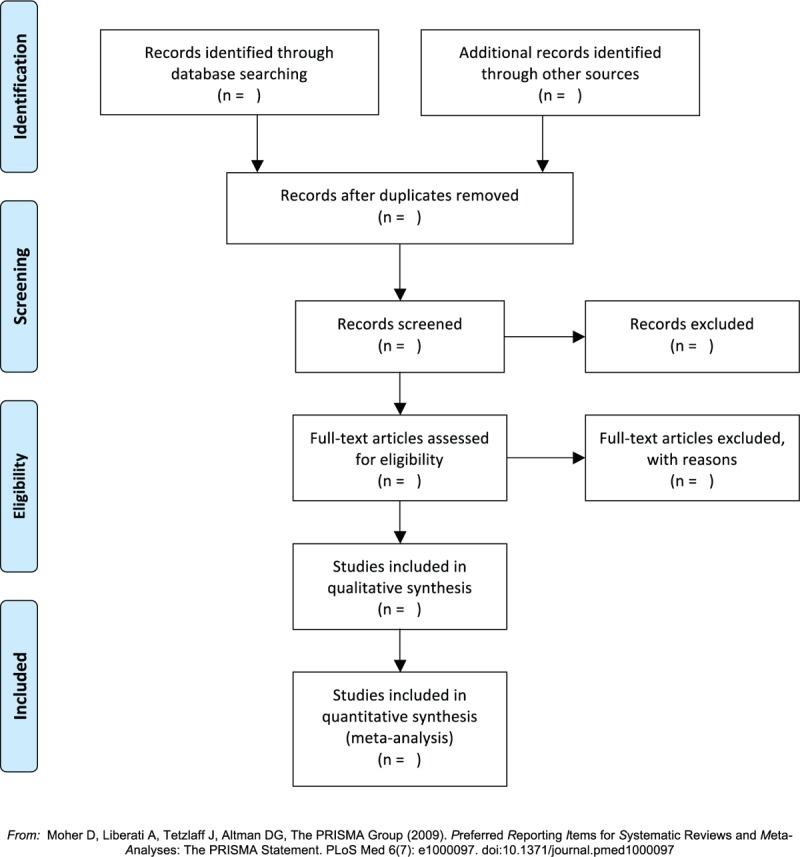
Flow diagram of studies search and selection.

This study will follow the guidelines outlined in the preferred reporting items for systematic reviews and meta-analysis (PRISMA) statement for meta-analyses of healthcare interventions.^[12]^ Additionally, the protocol adheres to the PRISMA Protocols (PRISMA-P).^[13]^ The selection process will be summarized according to PRISMA flow diagram (Fig. 1).

The protocol for this systematic review has been registered on PROSPERO under the number CRD42019131408.

### 3.2 Types of studies

Randomized controlled trials (RCTs) regarding intervention of artemisinin in ME associated with RVO will be included without restriction language.

### 3.3 Types of participants

Patients were histologically confirmed of RVO.

### 3.4 Types of interventions

Intervention: artemisinin therapy. Comparator: anti-VEGF drugs.

## 4 Search methods for the identification of studies

The Cochrane Library, MEDLINE, Embase, Chinese BioMedical Database (CBM), China National Knowledge Infrastructure (CNKI), Chinese VIP Information (VIP), Wangfang Database will be searched regardless of publication date, or language.

## 5 Data collection and analysis

### 5.1 Selection of studies and data extraction

Two reviewers will extract data independently from the eligible studies by using a standardized collection form. We will record the details of the eligible studies including: first author, publication time, study design, comparator, study period, numbers of outcomes, sex, age, type, histologic diagnosis, clinical pathological features, and outcomes. If above-mentioned information is not able to get, we will contact the corresponding author for detailed data. If there are discrepancies between the 2 reviewers, a final consensus will be resolved through discussion with the third researcher.

### 5.2 Assessment of risk of bias in included studies

We will use Cochrane Collaboration tool to assess the risk of bias.^[14]^ Each included study will be evaluated respectively by 2 researchers. Random sequence generation, allocation concealment, subjects and researchers blinded, outcome evaluation of blind method, the result data are incomplete and selective report results and other issues are involved and classified as “low,” “high,” or “unclear” based on Cochrane Collaboration tool. If there are divided opinions between 2 researchers in procession, we will resolve inconsistencies through discussion or asking for a help from a senior researcher.

### 5.3 Measures of treatment effect

We will apply relative risk (RR) to represent the enumeration data; measurement data will be represented by mean difference (MD) and 95% confidence interval (95% CI).

### 5.4 Dealing with missing data

Corresponding authors will be connected by E-mail for detailed data if their studies’ information is not available. If no additional message is received, we will conduct data synthesis using available data.

### 5.5 Assessment of quality in included studies

The quality of each selected studies will be evaluated using the Grading of Recommendations Assessment, Development and Evaluation (GRADE) approach by 3 investigators.

### 5.6 Assessment of heterogeneity

Random models will be applied to conduct the meta-analysis. We will use chi-squared-based *Q* test and *I*^2^ test to evaluate the heterogeneity of all studies included. And *P* < .10 or *I*^2^ values >50 means high heterogeneity among studies included.^[14]^ If there is a high heterogeneity, we will conduct subgroup analyses to explore the possible causes.

### 5.7 Assessment of reporting bias

If there are >10 included trials in this review, funnel plot will be used to discuss the reporting biases or small-study effects according Egger methods and Begger^[15]^ test with *P* < .05 indicating significant bias and a contribution of heterogeneity.

### 5.8 Data synthesis

We will use RevMan 5.3.5 software (The Cochrane Collaboration, Oxford, England, UK) and STATA 14 software (version 14.0; Stata Corp, College Station, TX) to calculate for data synthesis. If there no obvious statistical heterogeneity among the trails included, we will apply fixed effects model to perform in the analysis. However, the random effects model will be used, when apparent clinical heterogeneity among the trails included. Meanwhile, subgroup or sensitivity analysis will be conducted. *α* = 0.05 will be deemed statistically significant.

### 5.9 Subgroup analysis

Subgroup analysis will be conducted according to sex, histologic diagnosis, duration of artemisinin therapies, and anti-VEGF drugs.

### 5.10 Sensitivity analysis

Sensitivity analysis will be conducted to explore the quality of studies of the document following sample size, the outcome of missing data, and methodological quality.

### 5.11 Ethics and dissemination

Ethical approval is not required because individual patient information will be not used. The authors will disseminate this systematic review through conference presentations and peer-review publications.

## 6 Discussion

Expanding clinical applications of artimisinin is of interest to public health.^[16]^ Although, few studies have been reported to show the efficiency of artimisinin in the treatment of ME associated with RVO. With the further development of artemisinin research, there are more and more studies in this field. A systematic review which can provide the newest data should be conducted to show such therapeutic method associated with better VA and other examination results. Therefore, this protocol for a systematic review has to be displayed. We hope that our works will help clinicians with more convincing evidence about dealing with patients with ME secondary to RVO.

However, this review still has some limitations. Due to language barriers, only studies published in Chinese and English will be included. Besides, different countries and histology type of RVO may run risk of heterogeneity.

## Author contributions

Jing Xu and Xiaofeng Hao contributed to the conception of the study. Jing Xu, Xiaofeng Hao, and Bingwen Lu wrote the draft of manuscript, and was revised by Xiaoyu Li and Jing Ming. The search strategy was developed by all of the authors. Jing Xu, Xiaofeng Hao, and Yixin Qi will search, extract data, assess the risk of bias, and complete the data synthesis. Like Xie will arbitrate in case of disagreement and ensure the absence of errors. All authors approved the publication of the protocol.

**Conceptualization:** Jing Xu, Xiaofeng Hao.

**Data curation:** Jing Xu, Xiaofeng Hao, Xiaoyu Li.

**Funding acquisition:** Xiaofeng Hao, Yixin Qi.

**Methodology:** Jing Xu, Bingwen Lu.

**Project administration:** Jing Xu, Jing Ming.

**Supervision:** Like Xie.

**Validation:** Like Xie.

**Writing – original draft:** Jing Xu.

**Writing – review & editing:** Xiaofeng Hao, Bingwen Lu.
